#  Inadequate Evidence to Support Phase III Studies of Albumin in Severe Malaria

**DOI:** 10.1371/journal.pctr.0020004

**Published:** 2007-02-09

**Authors:** Charles J Woodrow, Timothy Planche

The study on severe malaria by Akech et al. [1] found no significant difference between albumin and Gelofusine in any outcome measure, yet the authors reject further investigation of Gelofusine and propose phase III studies of albumin as a neuroprotective agent potentially capable of reducing mortality in severe malaria by over 80% [2]. Several issues related to study design and interpretation require reconsideration of the authors' main conclusions.

Study design did not incorporate allocation concealment, a major omission that can influence patient recruitment at all stages from case finding strategy to consent [3]. Figure 1 gives the impression that patients were randomised after eligibility assessment, whereas in reality their treatment was known in advance. Comparison of baseline data for albumin and Gelofusine groups is an insensitive way to detect enrolment bias with these numbers of patients. An additional problem arises from a separate interventional study with phenytoin conducted simultaneously in comatose children, but no information is provided about possible interactions with fluid interventions. The task of undertaking two independent interventions in the same population would have benefited from prospective evaluation, for example, using a factorial design to minimise confounding.

Both intention to treat (ITT) and per protocol (PP) analyses are presented, with ITT including patients enrolled as an emergency who did not meet inclusion criteria. In isolation, this approach may appear reasonable but in similar previous studies Newton's group excluded such patients from all analyses. Given that two of four patients not meeting inclusion criteria but entering the Gelofusine arm subsequently died (versus zero of four in the albumin arm), has the decision to include such patients on this occasion been taken post hoc since it favoured albumin? The ITT analysis is quoted selectively at certain points, e.g., while Table 4 shows a mortality rate of 10% with Gelofusine (PP analysis), the ITT figure of 16% is reported in the text and press release. Table 3 describes deaths (eight for Gelofusine, not seven as reported throughout the text) without reference to patients failing inclusion criteria; we assume that these “out of criteria” deaths are cases 2 and 10. If so, the short time between admission and death (1 h and 3 h) suggests that these patients were moribund at presentation. The potential for bias to influence enrolment of these patients (see above), or the decision to include such patients in the ITT analysis, is evident.

Unfortunately, the editorial commentary compounds interpretative difficulties by incorrectly stating: “Death rates in hospital were lower in the group given albumin, and this was statistically significant.” Gelofusine was not associated with a significant increase in mortality compared to albumin (even by ITT analysis). Justification for albumin's superiority over Gelofusine is instead based on a small “meta-analysis” of studies of albumin versus “other fluids”, the dominant study of which enrolled 150 patients with 11 saline and two albumin deaths [4] (an alternative interpretation that large volumes of saline are hazardous has been discussed [5]). With only 80 patients enrolled in the Gelofusine versus albumin study, the “meta-analysis” was highly likely to generate the same result as its dominant study. We calculate that this albumin versus Gelofusine study could have had equivalent mortality in the two arms (up to 10 deaths per arm), yet the “meta-analysis” would still have showed significant benefit for albumin. Furthermore, there are discrepancies between the original studies and the “meta-analysis” both in total number of patients and deaths attributed to saline/Gelofusine (described, along with other inconsistencies, in our e-letter on the *PLoS Clinical Trials* Web site [http://clinicaltrials.plosjournals.org/perlserv/?request=read-response&doi=10.1371/journal.pctr.0010021#r1317]).

Based on these data, as well as preliminary studies of albumin as a neuroprotective agent in stroke, the authors now propose a phase III study with albumin, saline, and maintenance-only arms. This would again test two separate hypotheses simultaneously (volume resuscitation and brain protection), and ignores the possibility that saline may be dangerous [5]. If the authors are committed to studies of albumin as a neuroprotective agent, an appropriate development plan should include a prospective, randomised phase II trial of albumin versus maintenance-only fluid in patients with cerebral malaria, with specific monitoring for adverse events (particularly pulmonary oedema), as for studies of albumin in stroke [6].

The accompanying article providing support [7] for the authors' call for phase III studies has not clarified these issues. Complicated and important fields of research demand corresponding rigour. Phase III mortality studies ought to be based on appropriately designed and adequately powered phase II trials with close regard to safety.
